# A novel *AP4M1* mutation in autosomal recessive cerebral palsy syndrome and clinical expansion of AP-4 deficiency

**DOI:** 10.1186/s12881-014-0133-2

**Published:** 2014-12-14

**Authors:** Muhammad Jameel, Joakim Klar, Muhammad Tariq, Abubakar Moawia, Naveed Altaf Malik, Syeda Seema Waseem, Uzma Abdullah, Tahir Naeem Khan, Raili Raininko, Shahid Mahmood Baig, Niklas Dahl

**Affiliations:** Human Molecular Genetics Laboratory, National Institute for Biotechnology and Genetic Engineering (NIBGE), PIEAS, Faisalabad, 38000 Pakistan; Department of Immunology, Genetics and Pathology, Science for Life Laboratory, Uppsala University, Uppsala, 751 08 Sweden; Department of Radiology, Uppsala University, Uppsala, 751 85 Sweden

**Keywords:** Cerebral palsy, AP-4 deficiency, *AP4M1* gene, Mutation, Clinical variability

## Abstract

**Background:**

Cerebral palsy (CP) is a heterogeneous neurodevelopmental disorder associated with intellectual disability in one-third of cases. Recent findings support Mendelian inheritance in subgroups of patients with the disease. The purpose of this study was to identify a novel genetic cause of paraplegic CP with intellectual disability in a consanguineous Pakistani family.

**Methods:**

We performed whole-exome sequencing (WES) in two brothers with CP and intellectual disability. Analysis of *AP4M1* mRNA was performed using quantitative real-time PCR on total RNA from cultured fibroblasts. The brothers were investigated clinically and by MRI.

**Results:**

We identified a novel homozygous *AP4M1* mutation c.194_195delAT, p.Y65Ffs*50 in the affected brothers. Quantitative RT-PCR analysis showed markedly reduced *AP4M1* mRNA levels suggesting partial non-sense mediated mRNA decay. Several clinical and MRI features were consistent with *AP-4* complex deficiency. However, in contrast to previously reported cases with *AP4M1* mutations our patients show an aggressive behavior and a relatively late onset of disease.

**Conclusion:**

This study shows an *AP4M1* mutation associated with aggressive behavior in addition to mild dysmorphic features, intellectual disability, spastic paraparesis and reduced head circumference. Our findings expand the clinical spectrum associated with AP-4 complex deficiency and the study illustrates the importance of MRI and WES in the diagnosis of patients with CP and intellectual disability.

## Background

Cerebral palsy (CP) is a common cause of physical disability in childhood with an incidence of approximately 0.2-3% [1,2]. The causes of CP are heterogeneous and specific underlying causes remain unknown in the majority of cases. It is noteworthy that neonatal hypoxia, previously assumed to be a predominant cause of CP, accounts for no more than 10-20% of cases [3,4]. In line with this, independent studies have provided evidences for genetic causes behind CP related disorders. This was suggested already by an earlier twin-study showing an increased concordance rate for CP in monozygotic vs. dizygotic twins [5]. Furthermore, the association of CP with congenital malformations supported the hypothesis that genes may be involved in the disease [6,7]. More recent studies have confirmed that CP may be inherited as a Mendelian trait caused by single gene mutations in subgroups of patients. The four components of the adaptor protein 4 (AP-4) complex (reviewed by Moreno-De-Luca et al. [8]) have been linked to monogenic CP. The AP-4 hetero-tetramer is composed of the AP4B1, AP4M1, AP4E1 and AP4S1 subunits that are of critical importance for vesicular transport [9]. Thus, the AP-4 complex is required for appropriate intracellular transport as well as for secretion and endocytosis. Furthermore, the AP-4 complex sorts the AMPA glutamate receptors that are required for excitatory synaptic neurotransmission of importance during brain development [10]. To date, a limited number of families have been described segregating autosomal recessive CP that is caused by a mutation in either of the *AP4B1*, *AP4E1*, *AP4M1* and *AP4S1* genes [8,11-15]. The different mutations presumably cause a disruption of the AP-4 complex integrity and the affected individuals share clinical characteristics including intellectual disability, reduced head circumference, short stature and spastic di- or paraplegia. Symptoms such as hypotonia and/or microcephaly may be present at birth or in the neonatal period but development may be within normal ranges up to several months. The disease progresses usually within the first 2 years of age (y.o.a.) with loss of acquired motor functions, peripheral neuropathy, spasticity and sometimes seizures [14,15].

Here we report on a consanguineous family with two sons affected by a complex form of CP. The brothers had an onset at approximately 12 months of age (m.o.a) and they are homozygous for a novel and truncating *AP4M1* gene mutation associated with markedly reduced *AP4M1* mRNA levels. The clinical features in our patients bring further information on the variability and delineation of this subgroup of complex CP with AP-4 deficiency.

## Methods

### Patients

**Figure 1:**
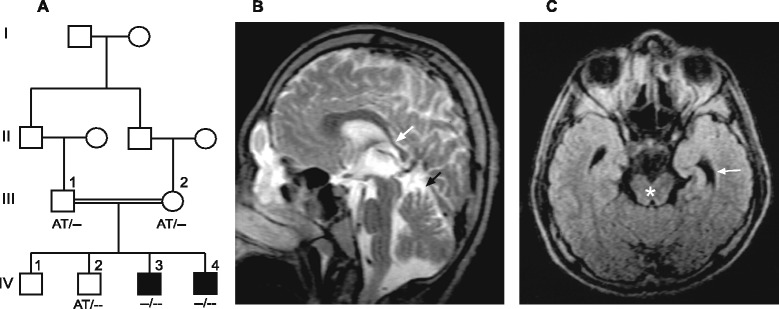
**Family structure and MRI findings. (A)** Pedigree of the consanguineous family with two affected brothers (filled squares). The *AP4M1* genotypes are indicated below the symbols. The AT nucleotides (c.194-195) represent the wild-type allele that is deleted (−−) on both alleles in the two affected brothers. **(B)** Sagittal T2-weighted MR image of ind. IV:3 shows a thin posterior third of the corpus callosum (white arrow). The upper vermis is slightly hypoplastic/atrophic (black arrow). **(C)** T1-weighted transverse image displays very wide temporal horns (arrow). The liquor containing space around the mesencephalon (*) is enlarged indicating slight dimensions of the brain stem and medial temporal lobes.

The consanguineous family is of Pakistani origin without family history of any neurological disease. The healthy parents are first cousins with four children (Figure 1A). Two sons have cerebral palsy, spastic paraplegia and intellectual disability whereas two sons are healthy. Informed and written consent was obtained from the parents being legal guardians of their sons. The study was carried out in accordance with the Declaration of Helsinki and the protocol approved by the local ethical committee, National Institute for Biotechnology and Genetic Engineering (NIBGE), >Faisalabad, Pakistan.

### Whole exome sequencing

Whole exome sequencing (WES) was performed on 50 ng of genomic DNA from the two affected brothers as described previously [16]. In brief, DNA was sheared by sonication with the Covaris S2 instrument (Covaris, Inc.). Fragment libraries were created from the sheared samples using the AB Library Builder System (Life Technologies) and target enrichment was performed according to the manufacturer’s protocols (Agilent SureSelect Human All Exon v4 kit). Exome capture was conducted by hybridizing the DNA libraries with biotinylated RNA baits for 24 h followed by extraction using streptavidin coated magnetic beads. Captured DNA was then amplified followed by emulsion PCR using the EZ Bead System (Life Technologies) and sequenced on the SOLiD5500xl system, generating over 100 million reads of 75 bp length for each of the samples.

Alignment of reads to the human reference sequence (hg19 assembly) and variant detection was performed using v2.1 of the LifeScope Software. SNPs and indel data was stored in an in-house exome database together with variant annotation information obtained from ANNOVAR [17] and dbSNP135. Custom R scripts were used to identify potentially damaging variants that were shared between the patients while not present in any of the other ~800 exomes in the in-house database.

### PCR and mRNA analysis

Segregation analysis of the *AP4M1* variant c.194_195delAT, p.Y65Ffs*50 was performed by PCR and Sanger sequencing using standard protocols. Total RNA was extracted from cultured fibroblasts of two patients and two control individuals using the PureLink RNA Mini kit (Invitrogen), treated with DNA-free (Ambion) and then converted into cDNA by using RevertAid H Minus First Strand cDNA Synthesis Kit (Fermentas). Quantitative real-time PCR was performed with the Platinum SYBR Green qPCR SuperMix-UDG kit (Invitrogen) then run and analyzed on the MxPro Real-Time PCR System (Stratagene). The primers for *AP4M1* mRNA quantification were designed to amplify exon-intron boundaries between exons 2 and 4, respectively, to ensure that the amplicons were cDNA specific. All reactions were performed three times and in triplicates and normalized to *β-actin* mRNA. Student’s two-tailed t-test was used for statistical analysis. Sanger sequencing was performed on the cDNA amplicon containing exon 3 in order to confirm the mutation. Primers sequences used to amplify the *AP4M1* cDNA and *AP4M1* exon 3 on genomic DNA were designed using Primer 3 Plus software (primer3plus.com/). All primer sequences are available upon request.

## Results

### Clinical investigation

Pregnancies and deliveries of both children were normal. Head circumference and muscle tonus were within normal ranges after delivery and during the first months of age. Hypotonia became evident in the elder brother (Ind. IV:3) at approximately 12 m.o.a and he learned to walk without support at 2 years of age. Spasticity developed gradually in the lower limbs with contractures of feet and positive Babinski sign. The ability to walk was lost at 6 years of age. Speech remained limited to a few single words and severe intellectual disability was evident at 3 years of age. He learned simple purposeful hand movements and he can feed himself. Seizures developed in childhood as well as an aggressive behavior in response to minor stimuli. Head circumference was -2SD at 14 years of age and facial dysmorphisms was otherwise restricted to a short philtrum (Table 1). MRI investigation, performed with a 0.23 T system at age 14 years of age showed a thin posterior corpus callosum, an enlarged third ventricle and widened temporal horns of the lateral ventricles. The mesencephalon and pons were thinner than expected for age. The upper vermis was hypoplastic/atrophic (Figure 1B-C). No abnormality in signal intensity was found.

**Table 1:** **Clinical features of patients with** ***AP4M1*** **mutations in this and previous studies**

**Parameters**	**Present study**		**Verkerk et al. [** 16 **]**	**Tüysüz et al. [** 15 **]**
	**Ind IV:3**	**Ind IV:4**		
Gender	M	M	3F/2M	3F/1M
Age at last examination (y.)	14	12	1.5/21/22/23/24	2.5/10.5/11/17
Head circumference	−2SD	−2SD	−1 to −2.5 SD	−2 to −4 SD
Height (cm)	157	137	NA	NA
Intellectual disability	Severe	Severe	Severe in 5/5	Severe in 3/4
Seizures	+	-	-	+
Shy character	-	-	+	+
Aggressive behavior	+	+	-	-
Stereotype laughter	-	-	+	+
Severe speech disorder	+	+	+	+
Infantile hypotonia	+	+	+	+
Hypertonia	+	+	+	+
Hyperreflexia	+	+	NA	NA
Babinski sign	+	+	+	NA
Spasticity	+	+	+	+
Club feet	-	-	+	+
Independent walking (y.)	2	4	-	+/−
Ambulation	-	-	-	-
*Craniofacial features*				
Facial hypotonia	-	-	NA	+
Bitemporal narrowing	-	-	NA	+
Broad nasal bridge	-	-	NA	+
Bulbous nose	-	+	NA	+
Short philtrum	+	+	NA	+
*Brain MR imaging*	Yes	No	3/5	4/4
Widened lateral ventricles	+		+	+
Thin splenium of the CC	+		+	+
Cerebellar hypoplasia/atrophy	+		2/3	-

*Abbreviations*: *+* present, *−* absent, *SD* standard deviation, *M* male, *F* female, NA: no data available, CC: corpus callosum, y.: years.

The second affected brother (ind. IV:4) was diagnosed with hypotonia at 12 months of age. Spasticity developed gradually in the lower limbs with contractures of feet and Babinski sign (Table 1). He learned to walk at 4 years of age but this ability was lost at 6 years of age. As for the elder brother, a severe cognitive deficit was evident with speech limited to a few single words, simple purposeful hand movement and aggressive behavior. Head circumference was -2SD at 12 years of age. Dysmorphic features included a short philtrum and a bulbous nose. The patient was not available for MRI investigation.

The upper limbs were unaffected in both brothers and none of them had pseudobulbar signs, impaired vision or hearing. Both brothers had a normal height, i.e. 157 cm and 137 cm at 14 years of age and 12 years of age, respectively.

### Whole exome sequencing

**Figure 2:**
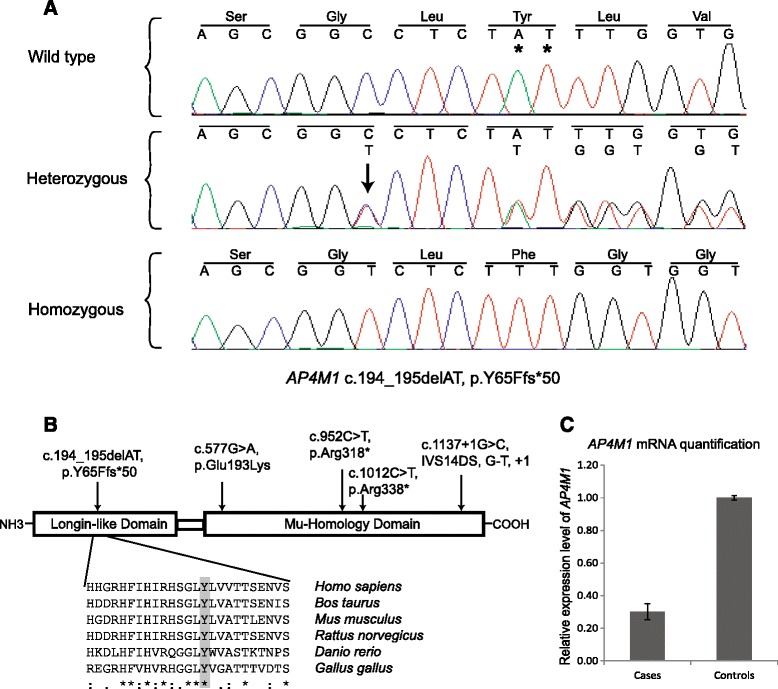
**Gene and expression analysis. (A)** Sequence chromatograms showing part of the *AP4M1* exon 3 (NM_004722.3) obtained from a healthy control (top), a heterozygous parent (middle) and the affected individual IV:3 (bottom). The two nucleotides in *AP4M1* (c.194_195delAT, p.Y65Ffs*50) deleted on the mutated allele are indicated by asterisks in the top panel. The arrow denotes a synonymous SNP (rs367614875) in the middle panel. Codons corresponding to the two alleles are indicated with the altered reading frame in the bottom chromatogram. **(B)** Schematic representation of the AP4M1 protein with its functional domains (http://www.ebi.ac.uk/interpro/protein/O00189) and all reported mutations (top). The degree of conservation of the Tyr65 residue (shaded) is shown across different species (bottom). **(C)** Quantitative RT-PCR of *AP4M1* mRNA expression in fibroblasts cells from cases (n = 2) and healthy controls (n = 2). Data are normalized to *β-actin* mRNA and presented as cases relative to controls ± standard deviation. The relative expression of *AP4M1* in controls is set to one. The *AP4M1* expression levels in patients are reduced three-fold when compared to controls (P = 0.0026, two sided Student’s t-test assuming equal variance).

Whole exome enrichment was performed on DNA samples from the two affected family members (ind. IV:3 and IV:4). After exome capture, the enriched DNA was sequenced using the Ion Proton system (Life technologies) and sequences were aligned to the human reference genome (hg19). An average of 97% of the exonic baits were covered at least 1x, and 90% were covered >10x. Common variants were excluded by filtering against dbSNP130 (MAF >0.01) and 800 in house exome. Using filtered WES data from the two brothers, we identified altogether nine novel candidate variants. Five missense variants were located in the X-chromosome genes *FANCB*, *CCNB3*, *RGAG1*, *GPR50* and *HAUS7*, respectively. However, none of the amino acid substitutions were predicted as pathogenic and the five genes were not previously associated with the clinical features observed in our patients. Four autosomal and homozygous variants were identified in the *TNFRSF14*, *AP4M1*, *RGMA* and *NINL* genes. One of these variants is located in exon 3 of the *AP4M1* gene (NM_004722.3) and consists of a two base pair deletion c.194_195delAT resulting in a frameshift p.Y65Ffs*50. With the previous knowledge on phenotypes associated with *AP4M1* we performed Sanger sequencing and confirmed homozygosity for the *AP4M1* deletion in the two affected family members. The unaffected parents and one healthy brother were heterozygous for the wild-type allele (Figure 2A). The eldest and healthy brother was not available for sampling. Furthermore, the two base pair deletion was excluded in 200 Swedish and 200 Pakistani control chromosomes and it was not present in *AP4M1* sequences from 800 in house exomes, the dbSNP (www.ncbi.nlm.nih.gov/SNP/) or the EVS data release (ESP6500SI-V2) on the Exome Variant Server, NHLBI GO Exome Sequencing Project (ESP), Seattle, WA (URL: http://evs.gs.washington.edu/EVS/) when accessed March 2014. The variant c.194_195delAT is located in the region encoding a highly conserved longin-like domain (Figure 2B). Longin domains are globular structures found in different protein families that mediate protein-protein interactions in the membrane trafficking machinery [18].

### mRNA analysis

To investigate the effect of the c.194_195delAT mutation at the transcript level we first sequenced the *AP4M1* cDNA amplicon spanning exon 3 from both brothers and we detected only the mutated transcript. We then quantified the *AP4M1* mRNA from cultured fibroblasts of the two affected family members (ind. IV:3 and ind. IV:4) as well as from control individuals using real-time qRT-PCR. The results indicated a 3-fold reduction in *AP4M1* mRNA expression in patient derived fibroblasts when compared to matched control fibroblasts (*p* < 0.0026, Figure 2C). The relative expression of *AP4M1* in cases with respect to controls where calculated after normalization to *β-actin*.

## Discussion

The phenotype in the two affected brothers of our family manifests as diplegic CP and severe intellectual disability. Autosomal recessive inheritance was likely because of the consanguineous and healthy parents. X-linked inheritance was possible but became highly unlikely when combining the results from bioinformatic analysis of X-chromosome variants with the clinical observations. Instead, a search for homozygous variants in our WES data revealed four autosomal genes including the *AP4M1* gene that is known to be associated with features observed in our patients. The *AP4M1* mutation c.194_195delAT mutation results in a frame-shift and a premature stop p.Y65Ffs*50. Further analysis of the *AP4M1* transcript in our patients showed that the deletion is associated with a three-fold reduction in *AP4M1* mRNA levels presumably because of incomplete non-sense mediated mRNA decay [19,20]. The frame-shift mutation is positioned within a region encoding the highly conserved longin-like domain located in the more N-terminal part of the protein. Longin domains are found in several protein families and serves as important regulators of membrane trafficking via protein–protein and intramolecular binding specificities [18]. Consequently, any translation of *AP4M1* c.194_195delAT mRNA predicts a protein with a loss of membrane trafficking properties as well as a complete loss of a functional C-terminal cargo-binding domain [21]. Thus, the truncated AP4M1 protein would most likely be non-functional even if integrated in the AP-4 complex and with a resulting loss of complex integrity. In combination, the previous reports on AP-4 complex deficiency together with our genetic and clinical findings made the *AP4M1* mutation a likely cause of the disease.

Our patients share several clinical features with other AP-4 deficient cases such as intellectual disability, reduced head circumference, spasticity and facial dysmorphisms, including a short philtrum and a bulbous nose [14]. Furthermore, the one individual available for MRI showed enlarged lateral ventricles and a thin corpus callosum that are typical findings reported previously in a few cases with mutations in each of the *AP4B1*, *AP4M1* and *AP4E1* genes, respectively [8,11-15]*.* In these previously reported patients signs of brain hypoplasia or atrophy are frequent but variable. Noteworthy, some clinical features in our patients were different when compared to the few previously reported cases with mutations in the *AP-4* subunit genes [8,11-15]. The clinical onset was relatively late, the body heights were within normal ranges for age and the upper limb functions were retained. Furthermore, the brothers have an aggressive behavior in response to minor stimuli in contrast to other patients with AP-4 deficiency. The reason for the clinical variation among CP patients carrying different mutations in *AP4B1*, *AP4M1*, *AP4E1* and *AP4S1* is unclear. It has been shown that the loss or structural change of a single AP-4 subunit disrupts the integrity of the entire AP-4 complex. Consequently, mutations in any of the AP-4 subunits would presumably have the same effects on vesicular glutamate receptor transport and neurotransmission resulting in similar clinical presentations. Therefore, one possible explanation for the clinical variability between individuals with mutations in genes encoding different AP-4 subunits is the effect from yet unknown modifier genes. This is further supported when comparing our observations with the two previously described families with truncating mutations in the *AP4M1* subunit gene [14,15].

Still, the AP-4 complex deficiency highlights two important aspects: It unravels a subgroup of patients with CP inherited as a Mendelian trait that can be recognized by a combination of appropriate diagnostic tools including WES. Second, AP-4 deficiency illustrates how a perturbed mechanism caused by interacting factors results in a clinical entity with CP and intellectual disability. In line with this, other factors and proteins involved in the AP-4 mediated vesicular trafficking may become strong candidates in the etiology of CP.

## Conclusions

We used whole-exome sequencing and identified a novel and truncating *AP4M1* mutation in two brothers with cerebral palsy and intellectual disability. Reduced levels of *AP4M1* mRNA in the patients suggest incomplete non-sense mediated decay. The clinical investigation revealed aggressive behavior, normal body height, retained upper limb function and relatively late onset that add to the clinical variability in AP-4 deficiency. Still, the combination of hypotonia in infancy, minor facial dysmorphisms, reduced head circumference or microcephaly, para- or tetraplegia, severe intellectual disability and typical MRI findings should make AP-4 deficiency a conceivable diagnosis. Our study provides additional support for autosomal recessive inheritance in a subgroup of patients with CP and intellectual disability. Further studies are now needed to establish the frequency of AP-4 mutations and their variable clinical outcome.

## Abbreviations

AP-4: Adaptor protein 4 complex
AP4M1: Adaptor protein 4 complex subunit M1
CP: Cerebral palsy
WES: Whole exome sequencing
MRI: Magnetic resonance imaging
RT-PCR: Reverse transcription polymerase chain reaction
AMPA: α-amino-3-hydroxy-5-methyl-4-isoxazolepropionic acid
cDNA: Complementary DNA
mRNA: Messenger RNA
SNP: Single nucleotide polymorphism
SD: Standard deviation
MAF: Mean allele frequency
